# Elastoplastic Indentation Response of Sigmoid/Power Functionally Graded Ceramics Structures

**DOI:** 10.3390/polym14061225

**Published:** 2022-03-17

**Authors:** Mohamed A. Eltaher, Ahmed Wagih, Ammar Melaibari, Ghazi S. Alsoruji, Mohamed A. Attia

**Affiliations:** 1 Mechanical Engineering Department, Faculty of Engineering, King Abdulaziz University, Jeddah 80204, Saudi Arabia; amelaibari@kau.edu.eg (A.M.); galsoruji@kau.edu.eg (G.S.A.); 2 Mechanical Design and Production Department, Faculty of Engineering, Zagazig University, Zagazig 44519, Egypt; awagih@zu.edu.sa (A.W.); maattia@zu.edu.sa (M.A.A.)

**Keywords:** bilinear TTO elastoplastic, sigmoid/power FG substrate, homogenization, finite element method, empirical indentation forms

## Abstract

Due to the applicability of new advanced functionally graded materials (FGMs) in numerous tribological systems, this manuscript aims to present computational and empirical indentation models to investigate the elastoplastic response of FG substrate under an indention process with spherical rigid punch. The spatial variation of the ceramic volume fraction through the specimen thickness is portrayed using the power law and sigmoid functions. The effective properties of two-constituent FGM are evaluated by employing a modified Tamura–Tomota–Ozawa (TTO) model. Bilinear hardening behavior is considered in the analysis. The finite element procedure is developed to predict the contact pressure, horizontal displacement, vertical deformation, and permanent deformation of FG structure under the rigid cylindrical indentation. The empirical forms for permanent deformation were evaluated and assigned. Model validation with experimental works was considered. The convergence of the mesh and solution procedure was checked. Numerical studies were performed to illustrate the influence of gradation function, gradation index, and indentation parameters on the contact pressure, von Mises stresses, horizontal/vertical displacements, and permanent plastic deformation. The present model can help engineers and designers in the selection of an optimum gradation function and gradation index based on their applications.

## 1. Introduction

At the end of the 20th century, the problems of singular stress distribution, stress peeling effects, and delamination observed in laminates’ structure were solved by Japanese’s scientists [1], who developed functional graded materials (FGMs). FGMs have continuous variation, from ceramic/metal phase to metal/ceramic content. Therefore, their material properties can be tailorable through applications [2]. FGMs can be used in various applications such as aerospace, marine, automobile, biomedical, defense, nuclear, turbine and MEMS/NEMS [3].

Different elastoplastic models were proposed to describe the elastoplastic response of FGMs by many researchers. In 1993, Williamson et al. and Drake et al. studied the elastoplastic response of FG cylindrical specimen geometries during a cooling process by using the finite element method (FEM). Akis [4] investigated the yielding and elastoplastic deformation of FG spherical vessels under internal pressure by using Tresca’s yield criteria and small deformation theory. Vaghefi et al. [5] implemented the von Mises yield criterion and isotropic strain hardening rule to consider the nonlinear elastoplastic behavior of FGM thick plate under the combination of mechanical and thermal loads using the meshless local Petrov–Galerkin method. Burzyński et al. [6] studied the elastoplastic nonlinear behavior of FGM shells under compression via Cosserat-type kinematics and FEM. Hasrati et al. [7] explored the elastoplastic postbuckling of thick plate by using Prandtl–Reuss and shear deformation Hencky constitutive relations. The stress–strain relation of the material was controlled by the Ramberg–Osgood elastoplastic model. Vakil and Zajkani [8] developed the lower order strain gradient model for analyzing the plastic behavior of FG crystalline microbeam structures. Vaghefi [9] studied the 3D thermo-elastoplastic bending response of FG skew plates subjected to combined thermal and mechanical loads by using von Mises isotropic hardening theory and the Prandtl–Reuss flow rule. Zhang et al. [10] developed the micromechanical model with experimental validation to predict the elastoplastic response of FGM manufactured by the vibration sedimentation process. Saeedi et al. [11] investigated the thermo-elastoplastic response of a thick FG cylindrical shell under internal pressure and temperature gradient by using the successive approximation method and differential quadrature method. Liu et al. [12] used a mesh-free reproducing kernel particle and penalty methods to study the elastoplastic behavior of the 2D structure with FGM composition.

Based on the Tamura–Tomota–Ozawa (TTO) elastoplastic model, Huang et al. [13,14] evaluated the buckling response of elastoplastic FGM cylindrical shells under torsional loads. Zhang et al. [15] studied the buckling behavior of elastoplastic FG cylindrical shells under combined compression and pressure. Nguyen et al. [16] examined the nonlinear bending of elastoplastic FGM beams under the influence of nonuniform distributed loads. Moita et al. [17] presented a nonlinear elastoplastic FG axisymmetric shell under thermal load based on a conical frustum FE model. Zhang et al. [18] investigated the thermal buckling of elastoplastic FGM Euler–Bernoulli beams under transversely nonuniform temperature rise. Zhu and Cai [19] developed an enhanced strain rate-dependent model to predict the elastoplastic response of FG composites under impact loads by using the Taylor dislocation model and Johnson–Cook model. All these studies demonstrated the efficiency of the TTO model to simulate the elastoplastic behavior of FGM.

Contact mechanics is presented in a wide variety of tribological systems whenever two bodies contact and roll over each other. Ma et al. [20] presented a normal contact stress, electric displacement and magnetic induction of 2D half-plane under a rigid flat/cylindrical punch by using the Fourier transform technique. Liu et al. [21] examined the contact pressure and deformation of FG substrate indented by rigid cylindrical/spherical punch by using the Hankel integral transform technique and the transfer matrix. Vasiliev [22] investigated the indentation of an electroelastic piezoelectric FG semi-infinite substrate by a rigid indenter under applied force and electric charge. Hou et al. [23] analytically investigated the stress gradient in the coated structure under tilted circular flat punch by using 3D elasticity theory. Asiri et al. [24] developed a predictive model to study the elastoplastic response during loading and unloading of nanocomposite structures indented by spherical punch. Chen and Yue [25] assessed the normal contact pressure between two elastic FGM spheres with different values of shear modulus and Poisson’s ratio. Wagih et al. [26] developed a numerical FEM to study the elastoplastic indentation of FGM substrate under a frictionless contact condition by using the TTO model. Melaibari et al. [27] presented a comprehensive numerical model to predict the deformation and contact force generated by indentation testing on the orthotropic microplates. All these studies were performed over either an orthotropic or isotropic FG plate following the power law gradation. Despite the importance of power law function in the gradation of FGM plates, the sigmoid function showed better stress transfer and a reduction in stress concentration within the thickness of the plate.

Considering the discussed state of the art, it was found that the gradation sigmoid function and elastoplastic contact have not been considered before. Hence, this article aimed to present a comprehensive analysis on the indentation behavior of sigmoid FG elastoplastic material, considering the gradation of all material parameters. Moreover, an empirical relation was developed to predict the permanent deformation in FG elastoplastic materials after indentation by knowing only the gradient index of the material, which will assist the engineer and scientist in their design. This article is organized as follows: Section 2 illustrates the gradation functions, rule of mixture, modified TTO model, bilinear hardening model, and homogenization procedure. Description of the problem, a finite element model and discretization techniques are presented in Section 3. Problem validation and mesh convergence are presented and discussed in Section 4. The numerical parametric studies and empirical permanent indentation form are illustrated and studied in Section 5. Section 6 summarizes the main points and conclusions of the present study

## 2. Elastoplastic Functionally Graded Material

**Figure 1:**
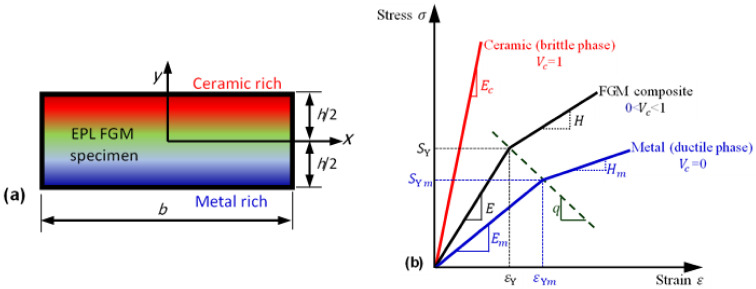
Typical stress–strain curve for a bilinear elastoplastic material FGM based on TTO model. (**a**) Coordinate system of material gradation, (**b**) Effective constitutive relation for FGM.

Consider an FGM specimen in the (x, y) coordinate system with width b and thickness h, as shown in Figure 1a. The material at the top and bottom surfaces of the specimen at y = ∓h/2 are made of pure metal (ductile phase) and pure ceramic (brittle phase), respectively. In this study, the spatial variation of the ceramic volume fraction, through the specimen thickness, is described using the power law (P-FGM) and sigmoid functions (S-FGM), respectively. The sigmoid–law distribution of FGM results in smooth stress distributions [28,29]. The volume fraction for S-FGM can be described by two power law functions [30]:

(1) $$V_{c}\left(y\right)=\left\{\begin{array}{l} \frac{1}{2}{\left(1+\frac{2y}{h}\right)}^{k}\;\;\;\;\;\;\;\;\;\;\;\;\;\;\;\left(-h/2\leq y\leq 0\right)\\ 1-\frac{1}{2}{\left(1-\frac{2y}{h}\right)}^{k}\;\;\;\;\;\;\left(0\leq y\leq h/2\right) \end{array}\right.;\;\;\;\;\;V_{c}+V_{m}=1$$

For P-FGM, the volume fraction is given as [30]:

(2) $$V_{c}\left(y\right)=1-{\left(\frac{1}{2}-\frac{y}{h}\right)}^{k}\;\;\;\;\;\;\;\;\left(-h/2\leq y\leq h/2\right);\;\;V_{c}+V_{m}=1$$

where the subscripts “*m*” and “*c*” refer to the metal and ceramic constituents, respectively, and $V_{i}$ is the volume fraction of the constituting phase *i*. k is the volume fraction index describing the material distribution.

The effective elastic properties of two-constituent FGM are evaluated employing the TTO model, i.e., modified rule of mixtures. The effective values of the modulus of elasticity $E\left(y\right)$ and Poisson’s ratio $\nu \left(y\right)$ can be expressed as [31]:

(3) $$E\left(y\right)=\left\{E_{m}\left(1-V_{c}\right)\frac{q+E_{c}}{q+E_{m}}+E_{c}V_{c}\right\}\times {\left\{\left(1-V_{c}\right)\frac{q+E_{c}}{q+E_{m}}+V_{c}\right\}}^{-1}$$

(4) $$\nu \left(y\right)=\nu_{m}\left(1-V_{c}\right)+\nu_{c}V_{c}$$

where $E_{i}$ and $\nu_{i}$ are, respectively, the Young’s modulus and Poisson’s ratio of the constituting material i. The stress transfer parameter q depends on the microstructure and material properties of FGM. To approximate the actual effects of microstructure interaction in FGMs, a nonzero finite value of q is used [32].

Following the TTO model, failure due to plastic deformation of a nonhomogeneous material having ductile and brittle phases is governed by the ductile constituent. During the plastic deformation of FGMs, the stress concentrations around the cracks and flaws of the ceramic phase are considerably reduced by the ductility and good shear strength of the metal phase and, thus, FGMs exhibit no brittle failure [32,33]. On the basis of this assumption, the effective yield strength $S_{Y}\left(y\right)$ and tangent modulus $H\left(y\right)$ of an elastoplastic FGM are evaluated using the stress transfer parameter as follows:

(5) $$S_{Y}\left(y\right)=S_{Ym}\left[\left(1-V_{c}\right)+\left(\frac{q+E_{m}}{q+E_{c}}\right)\frac{E_{c}}{E_{m}}V_{c}\right]$$

(6) $$H\left(y\right)=\left\{H_{m}\frac{q+E_{c}}{q+H_{m}}\left(1-V_{c}\right)+E_{c}V_{c}\right\}\times {\left\{\frac{q+E_{c}}{q+H_{m}}\left(1-V_{c}\right)+V_{c}\right\}}^{-1}$$

The overall elastoplastic behavior of FGMs is predicted by assuming a bilinear hardening behavior of the metal phase [33,34,35] (Figure 1b). The material properties of titanium–titanium boride (Ti/TiB) FGM, in which Ti and TiB represent the ductile metal and hard ceramic, respectively, are as follows: $E_{m}$ = 107 GPa, $E_{c}\;$= 3 75 GPa, $v_{m}$ = 0.34, $v_{c}$ = 0.14, $S_{Ym}$ = 450 MPa, $H_{m}$ = 14 GPa, and q = 4.5 GPa [13].

**Figure 2:**
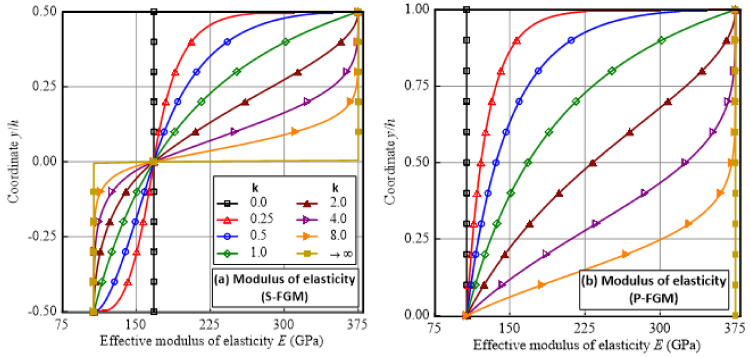

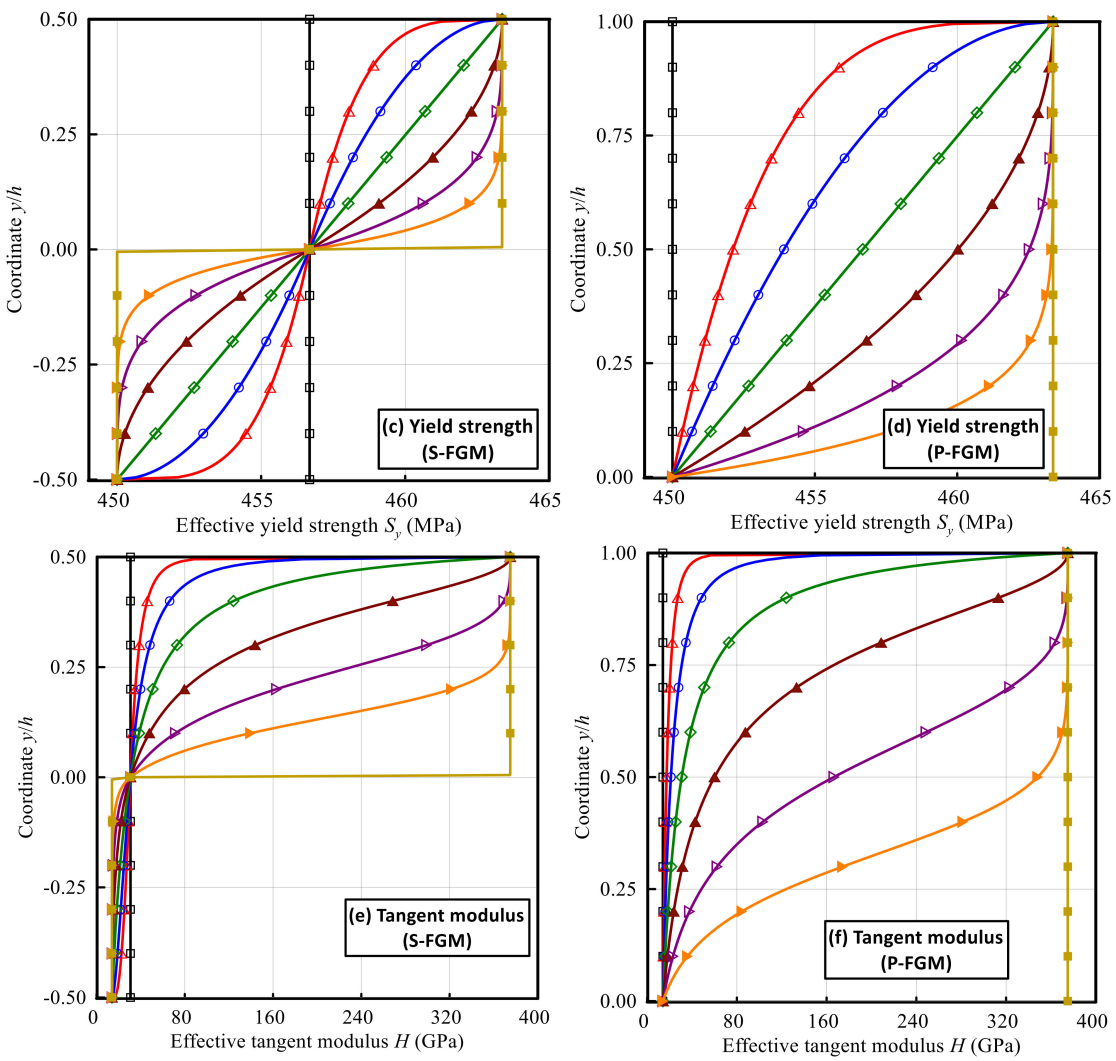
Variation in modulus of elasticity, yield strength, and the tangent modulus of the elastoplastic FGM Ti/TiB specimen along the thickness direction for the sigmoid function (S-FGM) and power law function (P-FGM). (**a**) Variation in modulus of elasticity of S-FGM, (**b**) Variation in modulus of elasticity of P-FGM, (**c**) Variation in yield strength of S-FGM, (**d**) Variation in yield strength of P-FGM, (**e**) Variation in tangent modulus of S-FGM, (**f**) Variation in tangent modulus of P-FGM.

Variations in the effective modulus of elasticity, yield strength, and tangent modulus of the Ti/TiB FGM specimen based on the sigmoid and power law functions are illustrated in Figure 2. It is depicted that for P-FGM, isotropic homogeneous specimens of pure metal and pure ceramic materials are recovered by setting k = 0 and k → ∞, respectively. On the other hand, the S-FGM specimen behaves as an isotropic homogeneous specimen at k = 0, with properties between those of Ti and TiB constituents. When k → ∞, the specimen exhibits a bi-material phase with metal and ceramic phases at the lower and upper half portions of the specimen, respectively. Thus, the transformation of the S-FGM from an isotropic homogeneous material phase to a bi-material phase is controlled by varying the gradient index k, as depicted in Figure 2a,c,e.

## 3. Finite Element Modeling

**Figure 3:**
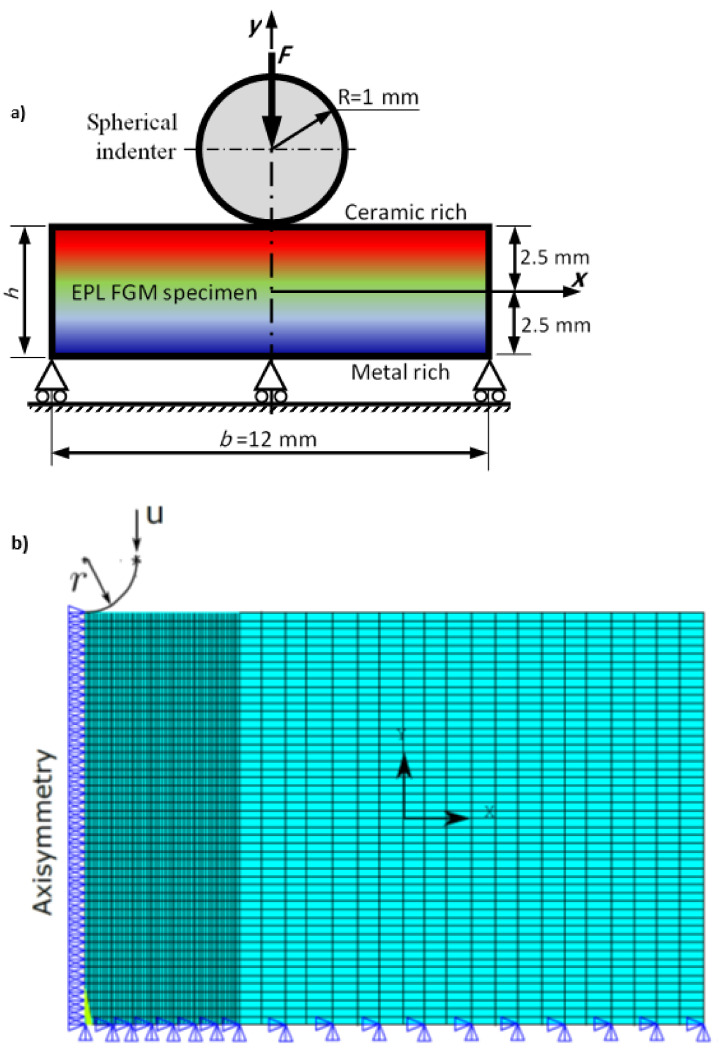
Schematic representation of the spherical indentation of an elastoplastic FGM specimen. (**a**) Representation of the spherical indentation problem (**b**) Presentation of boundary condition and meshing.

A 2D axisymmetric FE model on ANSYS software was implemented to simulate the indentation test experiment on functional graded structures. The FG substrate was defined as a rectangle of 12 mm width and 5 mm thickness, as shown in Figure 3a. Due to the axisymmetry of the material and indentation tests, half of the substrate was simulated with 6 mm width and 5 mm thickness and the axisymmetric boundary conditions were applied to the model at the left side of the substrate and the indenter geometries, as shown in Figure 3b.

The spherical indenter with 1 mm radius was defined as a rigid body to apply the indentation load. A frictionless contact pair was deployed to define the contact between the indenter and the specimen. The 2D structural plane elements (PLANE182) were used to mesh the substrate with graded mesh, where fine mesh was applied below the indenter and coarse mesh was applied to the rest of the substrate. The contact between the indenter and the substrate was defined as a surface-to-surface algorithm using contact (CONTA172) and target elements (TARGET169) for the contact pairs.

The augmented Lagrangian method was used as the contact algorithm. The elastic and plastic functional graded properties were applied using macro files where the constitutive Equations (1)–(6) were implemented including a loop to update the material properties of each element based on its position. The two gradation distributions with different gradation indices were implemented in the program using material code.

Fixed boundary conditions were defined for the nodes attached to the lower edge of the specimen. Displacement was applied to the pilot node of the indenter in the negative y-direction. The output of the simulation of the force–displacement response was recorded at the pilot node of the indenter. Four different meshes with different densities were deployed to determine the best mesh density to achieve accurate results with reasonable simulation time.

## 4. Validation and Convergence

**Figure 4:**
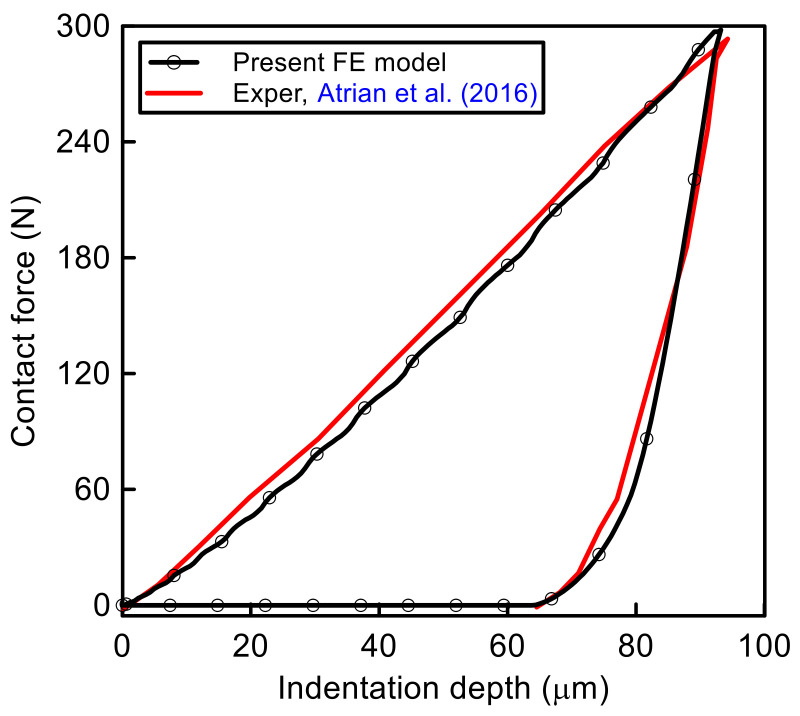
Validation of the present FE model with the experimental results of elastoplastic composite [36].

The validation of the simulation procedure of loading and unload indentation test with experimental results obtained by Artian et al. [36] is presented as shown in Figure 4. As illustrated, the numerical results obtained by FE are very close to the experimental test and a good agreement has been proven. During loading, the contact force was increased in the linear form to the maximum value of 300 N and indentation of 90 μm. After that, unloading was observed, and the contact force decreased linearly to 80 N and 82 μm indentation depth. The slope of the unloading was higher than the slope of the loading test. During unloading, after contact force of 80 N and indentation depth of 82 μm, the nonlinear phenomenon was observed. The permanent indention was recorded at 65 μm.

**Figure 5:**
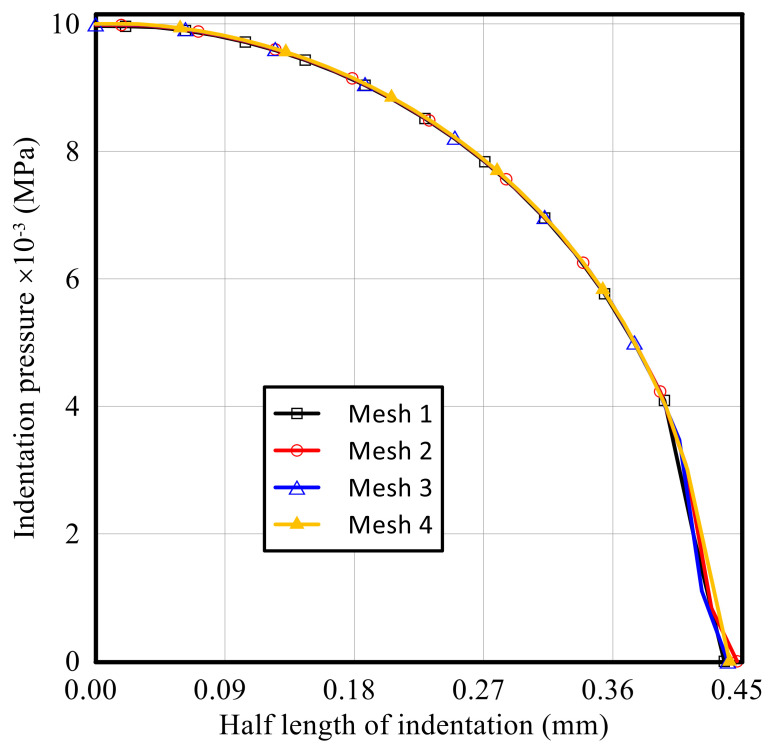
Influence of the FE mesh size on the distribution of the indentation pressure along the half length of indentation for an elastoplastic P-FGM Ti/TiB specimen at k = 0.5.

**Figure 6:**
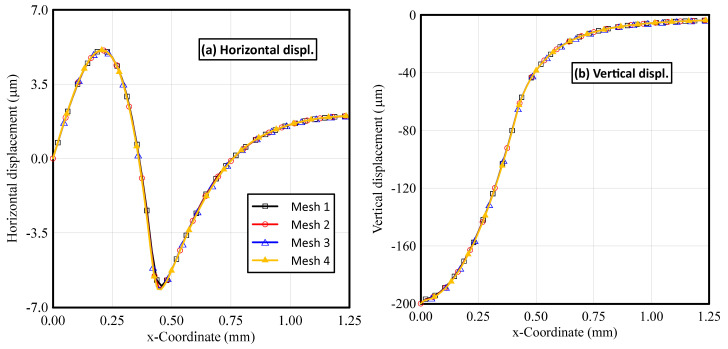

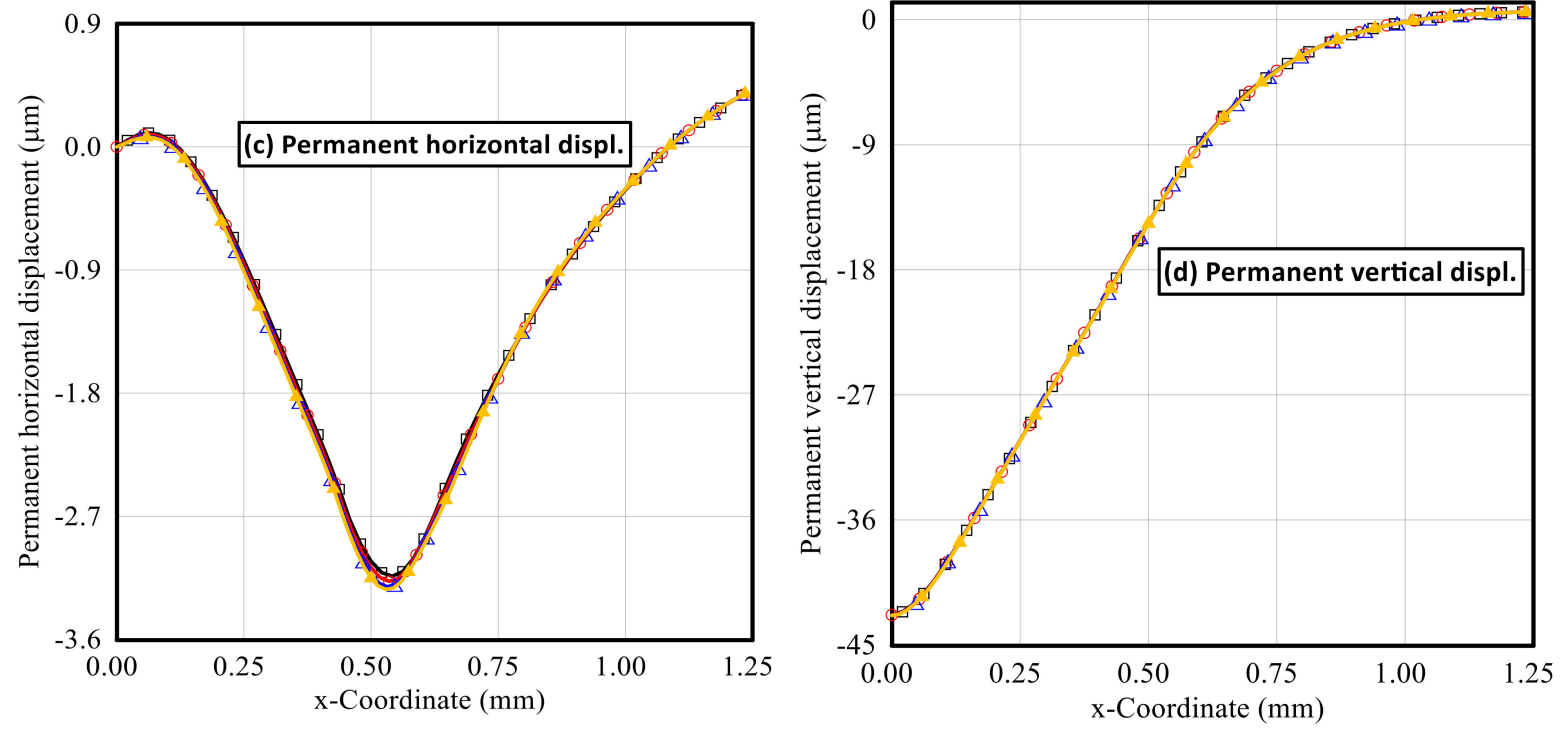
Influence of the FE mesh size on displacement profiles along the upper surface of an elastoplastic P-FGM Ti/TiB specimen at k = 0.5; (**a**) horizontal, (**b**) vertical, (**c**) permanent horizontal, and (**d**) permanent vertical displacements.

To demonstrate the accuracy and effectiveness of the finite element model, four different FE grids were constructed and compared. The proposed methodology was applied to investigate the FG elastoplastic indentation behaviors (i.e., indentation pressure, horizontal, vertical, permanent horizontal, and permanent vertical displacements) for an elastoplastic P-FGM Ti/TiB at gradation index *k* = 0.5, considering FE grids with different element size, as illustrated in Figure 5 and Figure 6.

As shown in Figure 5, four meshes are considered in the analysis: mesh 1 (60/5462) for 60 elements in the vertical direction and total elements in the domain of 5462; mesh 2 (70/6372); mesh 3 (80/8882); and mesh 4 (85/10,892). The maximum contact pressures for meshes 1, 2, 3 and 4 are 9.9664 × 10^−3^ MPa, 9.9836 × 10^−3^ MPa, 9.9617 × 10^−3^ MPa, and 9.5596 × 10^−3^ MPa, respectively, and corresponding contact areas are 0.4375, 0.4464, 0.44 and 0.4412. Therefore, the results of coarse meshes (meshes 1 and 2) are very close to each other, but deviate by 4.5% from the minimum indentation pressure observed in the case of mesh 4. The effects of mesh size on the horizontal and vertical displacements are portrayed in Figure 6. It is concluded that by refining the mesh, the horizontal and vertical displacements are changed. The horizontal displacement changed from 6.0071 µm at mesh 1 to 6.1623 µm at mesh 2; the permanent horizontal displacement changed from 3.139 µm to 3.2354 µm; the vertical displacement changed from 197.32 µm to 198.03 µm; and permanent vertical displacements changed from 42.825 µm to 42.884 µm. From Figure 5 and Figure 6, it can be proved that the response predicted from the present numerical simulation is very close to the experimental data, and the numerical results are convergent.

## 5. Numerical Results and Discussions

In this section, the applicability of the proposed numerical methodology to investigate the nonlinear indentation response of elastoplastic FG materials is demonstrated. The effects of material characteristics distribution throughout the thickness direction are investigated, considering both S-FGM and P-FGM. The mechanical indentation behavior is obtained for the two different distributions at different values of the material gradation index. The normalized penetration depth is defined as $∆=\frac{U_{y}}{U}$, with $U_{y}$ being the resulting vertical displacement while U is the applied prescribed vertical displacement.

**Figure 7:**
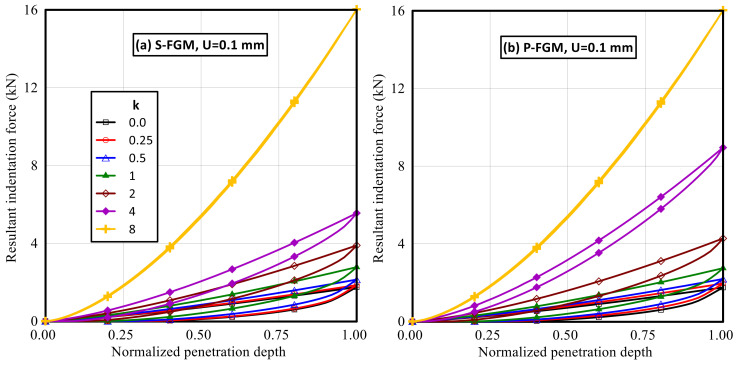
Indentation force–penetration depth curves for the elastoplastic Ti/TiB specimen at different gradient indices; (**a**) S-FGM, and (**b**) P-FGM.

Variations of the resultant indentation force versus the normalized penetration depth for both S-FGM and P-FGM at different material gradation index are illustrated in Figure 7. It is demonstrated that the indentation force required to attain a certain indentation penetration depth is governed by the material gradation index for both S-FGM and P-FGM distributions. In general, increasing the material gradation index decreases the material flexibility, which increases the required indentation force for a certain indentation depth. Additionally, increasing the material gradation index increases the ceramic content of FGM, which decreases the enclosed loop between loading and unloading paths, leading to elastic response at higher values of *k* (*k* = 8). It is observed that larger values of the resultant indentation force are required to attain a certain indentation penetration depth for P-FGM compared with the corresponding S-FGM distributions, especially at *k* = 2 and *k* = 4. Moreover, enclosed loops with a larger size between loading and unloading paths are observed for S-FGM compared with the corresponding cases for P-FGM due to its higher flexibility.

**Figure 8:**
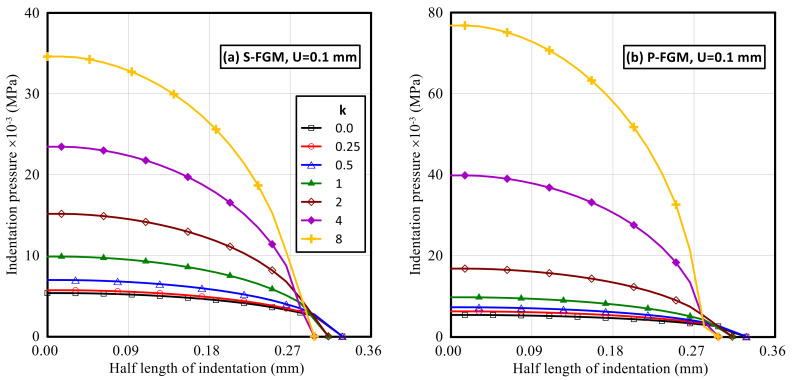
Distributions of the indentation pressure (at full load) along the half length of indentation for the elastoplastic Ti/TiB specimen at different gradient indices; (**a**) S-FGM, and (**b**) P-FGM.

The indentation pressure profiles throughout the indentation interface for both S-FGM and P-FGM distributions for different material gradation indices are shown in Figure 8. It is revealed that the indentation pressure increases when increasing the material gradation index for both material distributions due to the increase in material stiffness. Additionally, for the same indentation depth, larger values of the resulting indentation pressure are produced with P-FGM compared with S-FGM. This effect is most significant at a material gradation index for *k* > 1.

**Figure 9:**
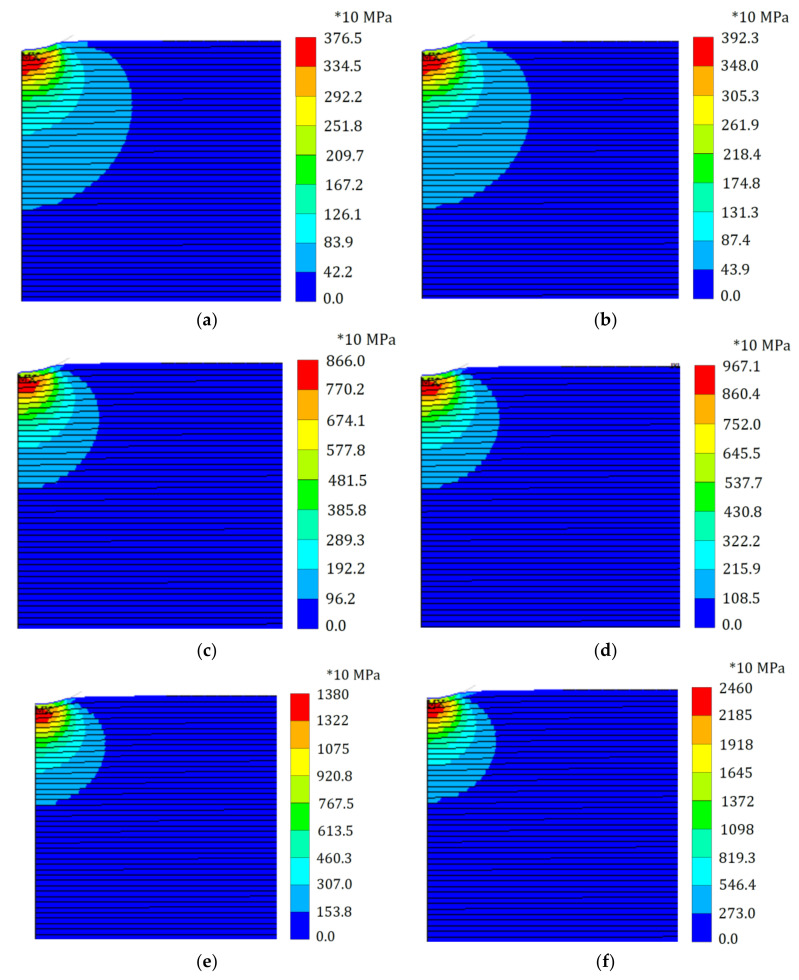
Distributions of the von Mises equivalent stress (at full load) within the elastoplastic Ti/TiB specimen at different gradient indices; (**a**) S-FGM with K = 0.5, and (**b**) P-FGM with K = 0.5, (**c**) S-FGM with K = 2, (**d**) P-FGM with K = 2, (**e**) S-FGM with K = 4 and (**f**) P-FGM with K = 4.

**Figure 10:**
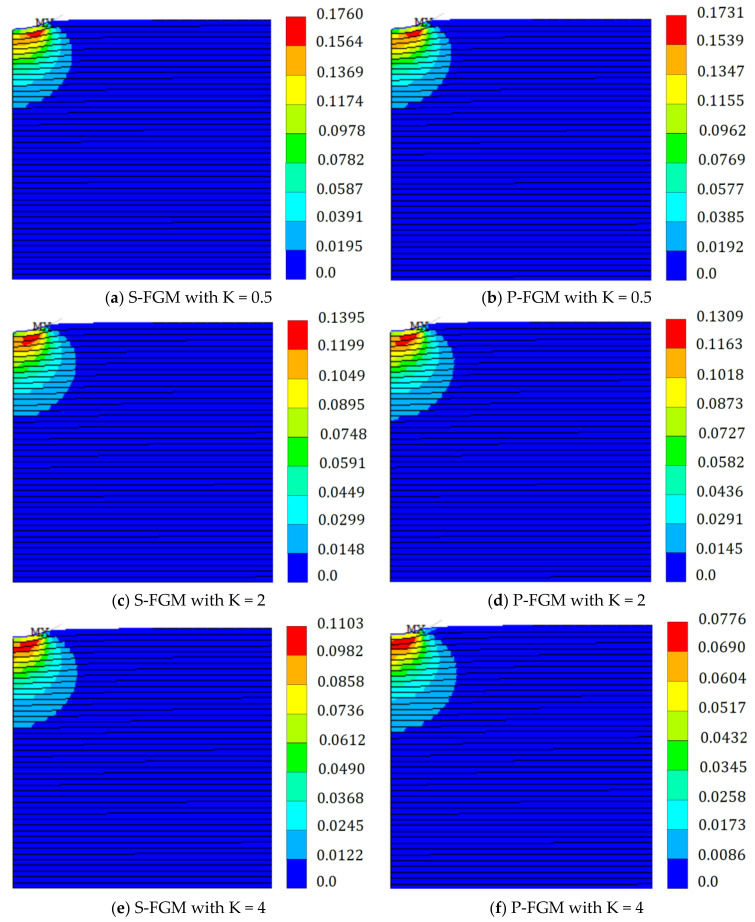
Distributions of the equivalent plastic strain (at full load) within the elastoplastic Ti/TiB specimen at different gradient indices; (**a**) Equivalent plastic strain of S-FGM at K = 0.5, and (**b**) Equivalent plastic strain of P-FGM at K = 0.5. (**c**) Equivalent plastic strain of S-FGM at K = 2, and (**d**) Equivalent plastic strain of P-FGM at K = 2. (**e**) Equivalent plastic strain of S-FGM at K = 4, and (**f**) Equivalent plastic strain of P-FGM at K = 4.

Distributions of the equivalent von Mises stress and the equivalent plastic strain at full load within the elastoplastic Ti/TiB specimen at different gradient indices for both S-FGM and P-FGM distributions are depicted in Figure 9 and Figure 10, respectively. It is observed that increasing the material gradation index through the loading path results in increasing the equivalent von Mises stress and decreasing the equivalent plastic strain due to decreasing the material compliance for both S-FGM and P-FGM distributions. On the other hand, due to the increase in S-FGM compliance, smaller values of the equivalent von Mises stress and larger values of the equivalent plastic strain are produced compared with the corresponding cases of P-FGM for all values of the material gradation index.

**Figure 11:**
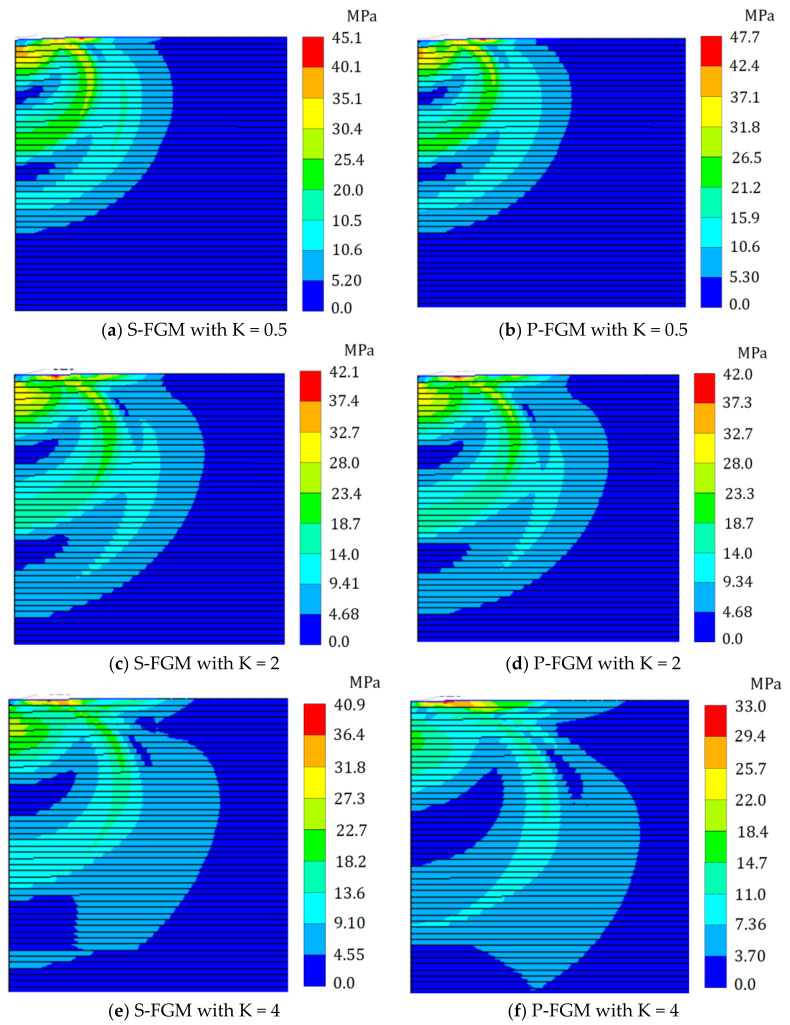
Distributions of the residual von Mises equivalent stress (after unloading) within the elastoplastic Ti/TiB specimen at different gradient indices; (**a**) the residual von Mises equivalent stress of S-FGM at K = 0.5, and (**b**) the residual von Mises equivalent stress of P-FGM at K = 0.5. (**c**) the residual von Mises equivalent stress of S-FGM at K = 2, and (**d**) the residual von Mises equivalent stress of P-FGM at K = 2. (**e**) the residual von Mises equivalent stress of S-FGM at K = 4, and (**f**) the residual von Mises equivalent stress of P-FGM at K = 4.

The map of the residual von Mises equivalent stress after unloading within the elastoplastic Ti/TiB specimen at different gradient indices for both S-FGM, and P-FGM distributions is shown in Figure 11. It is depicted that the residual von Mises equivalent stress after unloading decreases when increasing the material gradation index due to the increase in the overall system stiffness for both material distributions. Additionally, larger values of the residual von Mises equivalent stress are detected for P-FGM at *k* = 0.5 compared to those obtained by S-FGM. For *k* = 2 and 4, the response is reversed, and larger values of the residual von Mises equivalent stress are induced in S-FGM compared with the corresponding cases of P-FGM.

**Figure 12:**
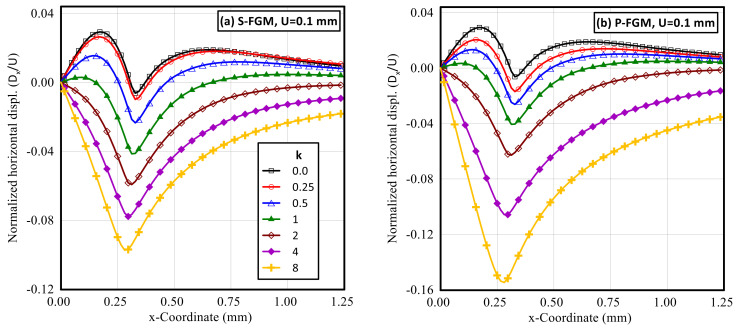
Profiles of the horizontal displacement (at full load) along the upper surface of an elastoplastic Ti/TiB specimen at different gradient indices; (**a**) S-FGM, and (**b**) P-FGM.

**Figure 13:**
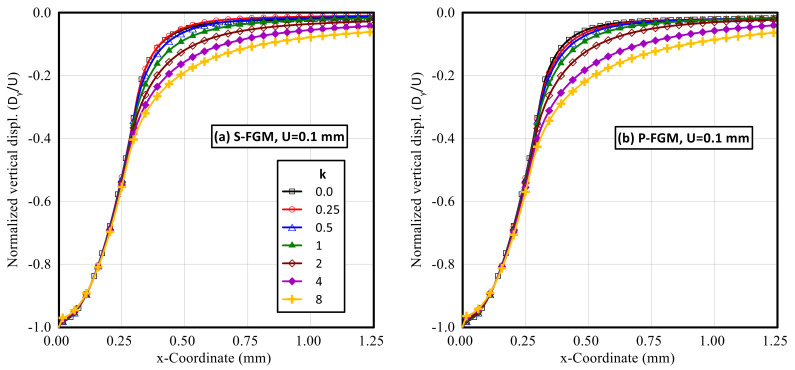
Profiles of the vertical displacement (at full load) along the upper surface of an elastoplastic Ti/TiB specimen at different gradient indices; (**a**) S-FGM, and (**b**) P-FGM.

Both normalized horizontal and vertical displacement profiles along the upper surface of the elastoplastic FG indented specimen at different gradient indices for both S-FGM and P-FGM distributions at full load are shown in Figure 12 and Figure 13, respectively. It is demonstrated that the absolute value of the induced displacement increases with increase in the material gradient parameter for both material distributions. Due to the nature of the applied load and prescribed vertical displacement, the effect of the material gradient parameter on the absolute value of the vertical displacement was insignificant. Comparing the two material distributions, larger values of the induced displacements are produced by the P-FGM due to its lower compliance.

**Figure 14:**
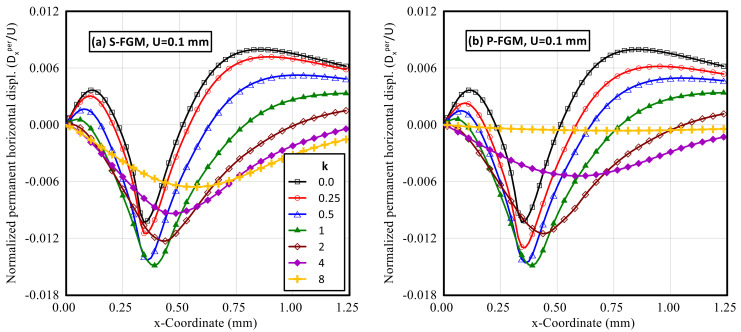
Profiles of the permanent horizontal displacement (after unloading) along the upper surface of an elastoplastic Ti/TiB specimen at different gradient indices; (**a**) S-FGM, and (**b**) P-FGM.

**Figure 15:**
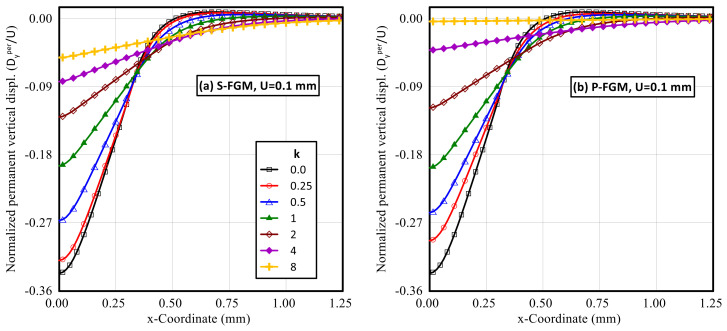
Profiles of the permanent vertical displacement (after unloading) along the upper surface of an elastoplastic Ti/TiB specimen at different gradient indices; (**a**) S-FGM, and (**b**) P-FGM.

After unloading, the induced horizontal and vertical permanent displacements were significantly affected by the material distributions. Profiles for the normalized horizontal and vertical permanent displacements throughout the upper surface of the functionally graded indented specimen at different gradient indices for both S-FGM, and P-FGM distributions after unloading are illustrated in Figure 14 and Figure 15, respectively. It is observed that for 0 < *k* ≤ 1, the absolute value of the normalized permanent horizontal displacement increases when increasing the material gradient index for both material distributions while, for *k* > 1, the absolute value of these permanent horizontal displacements decreases with increasing k. On the other hand, the absolute value of the normalized permanent vertical displacement increases with the increase in material gradient index.

Comparison of the two material distributions revealed that for the same vertical indentation depth, larger values of the absolute permanent displacements are detected by the S-FGM due to its higher compliance while, at higher values of the material gradient index, *k* = 8 complete recovery is attained by the P-FGM and reached zero permanent displacements.

The investigated mechanical behaviors of the elastoplastic FG indentation behavior for both S-FGM and P-FGM distributions at indentation depths 0.1 and 0.2 mm are summarized in Table 1. It is demonstrated that both the total and elastic strain energies increase with increases in material gradient index and indentation depth for both material distributions. Additionally, larger values of these strain energies are produced for P-FGM compared with the corresponding values obtained for S-FGM. On the other hand, the dissipation energy due to the plastic deformation is significantly affected by the material distributions. For *k* = 0.25, 0.5, 1.0, and 2.0, the dissipated energy increases with the increase in material gradient index and decreases for *k* = 4 and 8. Additionally, for all material gradient indices, the S-FGM distribution produces larger values for dissipative energy than those induced in the P-FGM distribution.

Based on the obtained numerical results, the following demonstrate empirical relations for the permanent depth with the material gradient index.

(7) $$\;{\bar{U}}_{max}^{per}=\frac{{\left.U_{max}^{per}\right|}_{FGM}}{{\left.U_{max}^{per}\right|}_{Metal}}={\left(\frac{1}{{\left[1.5\left(k^{0.14}\right)\right]}^{m}+{\left[0.17\left(k^{2.6}\right)\right]}^{m}}\right)}^{\frac{1}{m}},\;\;m=0.92\;\;\;\;\;\;\;\;\;\;P-FGM\;$$

(8) $${\bar{U}}_{max}^{per}=\frac{{\left.U_{max}^{per}\right|}_{FGM}}{{\left.U_{max}^{per}\right|}_{Metal}}={\left(\frac{1}{{\left[1.13\left(k^{0.06}\right)\right]}^{n}+{\left[0.15\left(k^{1.4}\right)\right]}^{n}}\right)}^{\frac{1}{n}},\;\;n=0.6\;\;\;\;\;\;\;\;\;\;\;\;\;\;S-FGM\;$$

where *m* and *n* are material-dependent parameters, which depend on the properties of the considered elastoplastic functionally graded material [37], incorporating the gradation of all the material properties.

**Figure 16:**
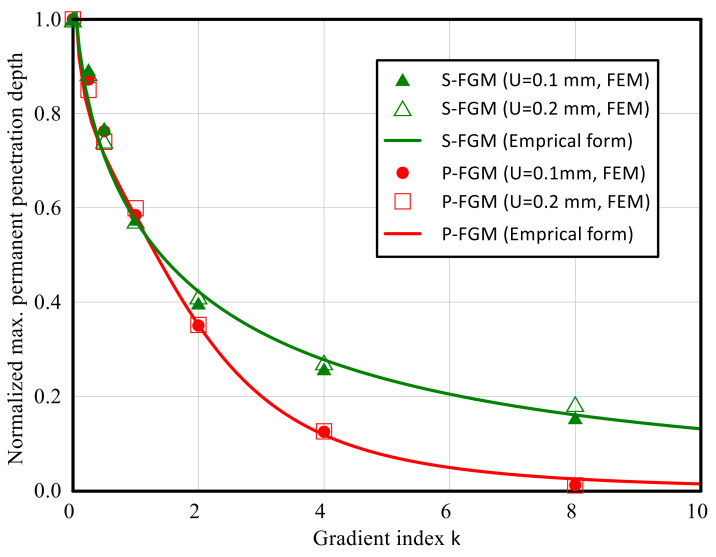
Prediction of the permanent indentation depth as a function of the gradient index for both S-FGM and P-FGM functions of elastoplastic Ti/TiB specimen, accounting for the gradation in all material properties.

The dependency of the normalized maximum permanent penetration depth on the material gradient index is investigated using the proposed numerical procedure and the empirical formula (Equations (7) and (8)) for two different indentation depths: *U* = 0.1 and 0.2 mm. As shown in Figure 16, it can be observed that a good correlation is obtained between the predictions using the empirical formulas illustrated in Equations (7) and (8) with material parameters *m* = 0.92 and *n* = 0.6 and the FE results. Consequently, using the obtained empirical formulas and evaluating the material parameters m and n, the indentation depth of an elastoplastic functionally graded material with any gradation index could be predicted with no need for computational experimental efforts.

**Figure 17:**
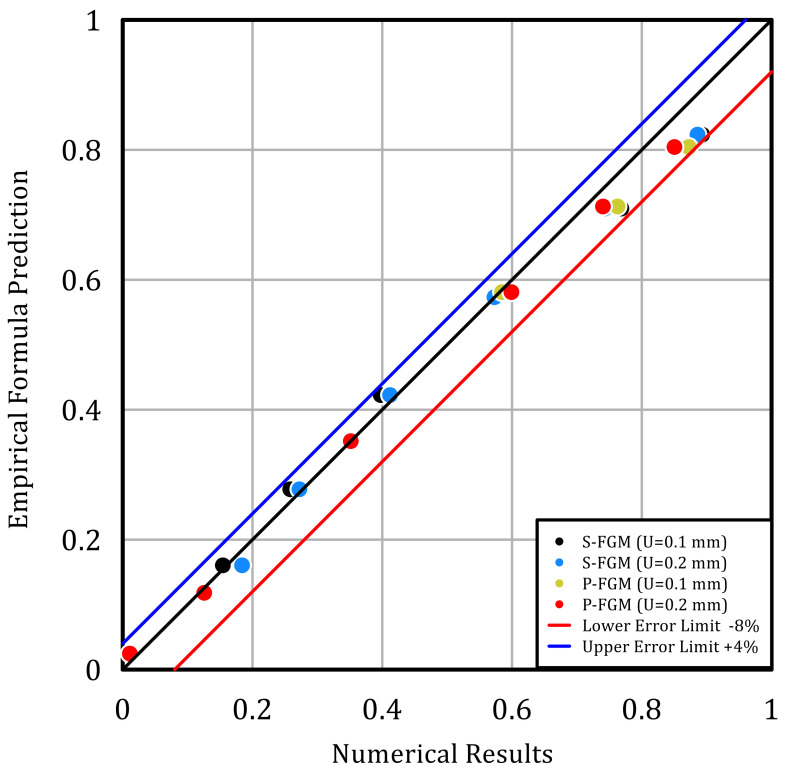
Normalized max permanent penetration depth (empirical) vs. normalized max permanent penetration depth (numerical).

To illustrate the effectiveness of the obtained empirical formulas, the maximum and minimum errors between the investigated normalized maximum permanent penetration depth by the empirical equations and the corresponding results obtained by the FE are illustrated in Figure 17. It is seen that the maximum error, ℇ, is bound between −8 < ℇ < 4, validating the effectiveness of the obtained empirical formulas for both S-FGM and P-FGM distributions.

## 6. Conclusions

The nonlinear mechanical behavior of elastoplastic FGM under spherical indentation is studied and analyzed considering two different material distributions. Both the power and sigmoid functions are exploited to express the spatial variation of the ceramic volume fraction throughout the indented FGM thickness direction. The modified Tamura–Tomota–Ozawa (TTO) model is adopted to describe the elastoplastic FGM distribution. A computational finite element procedure is exploited to investigate the nonlinear mechanical indentation behavior. The computational methodology is checked and verified. Numerical results are obtained and discussed. Based on the obtained numerical results, the dependency of the normalized maximum permanent indentation depth on the material gradient index is empirically obtained for the considered FGM. The results obtained revealed that:

- S-FGM distribution results in more compliant material than P-FGM distributions. Thus, a smaller indentation force and a larger enclosed loop size between the loading and unloading path are predicted, especially for gradient index *k* > 2.
- The von Mises equivalent stress at full was significantly affected by the material gradient index; it is increased by increasing the material gradient index for both S-FGM and P-FGM distributions.
- The von Mises residual stresses greatly affected by the material distributions and their gradient index. These residual stresses are increased with the increase in gradient index for both S-FGM and P-FGM distributions for 0 < *k* < 1.0 and decrease for *k* > 2.0.
- The total and elastic strain energies are dependent on both material distributions and their gradient indices. These energies are increased with the increase in gradient index for both S-FGM and P-FGM distributions. Additionally, for the same indentation depth, larger energy values are induced for P-FGM than those produced by S-FGM for all values of the material gradient indices.
- The dissipated energy due to the plastic deformation was significantly affected by the FGM distributions and their gradient index. This dissipated energy increases with the increase in gradient index for *k* = 0.25, 0.50, 1.0, and 2.0 and decreases with the increase in gradient index for *k* > 2 for both S-FGM and P-FGM distributions. Due to the increase in material compliance of S-FGM, a larger amount of dissipative energy is detected compared with the corresponding cases for P-FGM distributions.
- In general, the FGM characteristics’ distribution throughout the thickness direction significantly affects the mechanical behavior of the elastoplastic material under spherical indentation. This mechanical behavior could be controlled by selecting the suitable material distribution law with a controlled material gradient index.
- The study can be extended for different elastoplastic materials with a wide range of properties to generalize the developed empirical equation, so that the response of the general elastoplastic sigmoid FGM became well understood and predictable using these empirical relations.

## Figures and Tables

**Table 1:** Summary of the different indentation variables at an applied displacement U = 0.1 and 0.2 mm.

Variable	U (mm)	k = 0		k = 0.25		k = 0.5		k = 1.0		k = 2.0		k = 4.0		k = 8.0	
		P-FGM	S-FGM	P-FGM	S-FGM	P-FGM	S-FGM	P-FGM	S-FGM	P-FGM	S-FGM	P-FGM	S-FGM	P-FGM	S-FGM
**Max. indentation pressure/10^3^ (MPa)**	0.1	5.349	5.349	6.232	5.706	7.244	6.965	9.710	9.864	16.788	15.150	39.800	23.450	76.800	34.606
	0.2	7.252	7.252	8.526	7.805	9.994	9.620	13.581	13.794	23.945	21.419	58.527	33.460	116.670	49.537
**Max. indentation force (kN)**	0.1	1.751	1.751	1.957	1.850	2.188	2.137	2.744	2.777	4.275	3.892	8.954	5.552	16.030	16.030
	0.2	4.348	4.348	4.920	4.662	5.570	5.456	7.130	7.218	11.480	10.280	25.160	14.820	47.060	20.340
**Max. permanent** **horizontal displacement (µm)**	0.1	−1.032	−1.032	−1.301	−1.148	−1.460	−1.432	−1.489	−1.489	−1.152	−1.230	−0.542	−0.939	−0.063	−0.658
	0.2	−3.129	−3.129	−3.246	−3.015	−3.215	−3.136	−2.912	−2.902	−2.052	−2.269	−0.996	−1.831	−0.078	−1.814
**Max. permanent vertical displacement (µm)**	0.1	−33.70	−33.70	−29.38	−32.00	−25.67	−26.70	−19.74	−19.48	−11.83	−13.02	−4.17	−8.34	−0.39	−5.19
	0.2	−56.56	−56.56	−49.09	−53.09	−42.87	−44.02	−32.86	−32.42	−19.90	−22.27	−7.60	−15.33	−0.62	−12.42
**Max. von Mises stress (10 MPa)**	0.1	280.1	280.1	332.3	303.4	392.3	376.5	538.4	548.9	967.1	866.0	2460	1380	5342	2456
	0.2	394.2	394.2	469.5	428.6	556.5	535.4	771.7	782.5	1405	1240	3642	1972	3645	3001
**Max residual von Mises stress (10 MPa)**	0.1	38.6	38.6	38.9	39.6	42.4	45.1	43.3	42.2	42.0	42.1	33.0	10.9	7.51	4.33
	0.2	436.2	436.2	45.7	43.9	47.2	46.5	49.0	48.6	47.8	48.4	37.4	45.4	37.3	42.2
**Effective plastic strain**	0.1	0.1898	0.1898	0.1875	0.1812	0.1731	0.1760	0.1582	0.1573	0.1309	0.1349	0.0776	0.1104	0.0103	0.0985
	0.2	0.2727	0.2727	0.2587	0.2701	0.2051	0.2515	0.2238	0.2229	0.1870	0.1913	0.1151	0.1574	0.1151	0.1210
**Total energy (N × mm)**	0.1	75.69	75.69	83.95	79.60	93.28	91.13	115.54	116.88	176.37	161.56	358.78	227.82	626.67	626.67
	0.2	376.07	376.07	422.36	399.72	474.66	464.06	600.04	607.58	947.29	856.98	2021.03	1227.98	3687.05	1694.68
**Elastic energy (N × mm)**	0.1	30.64	30.64	37.46	33.10	45.41	43.08	65.11	66.35	122.95	109.18	313.55	176.10	618.93	618.93
	0.2	167.39	167.39	206.54	184.45	251.98	241.08	364.13	370.77	691.95	605.71	1786.40	965.82	3654.89	1410.82
**Plastic dissipative** **energy (N × mm)**	0.1	45.047	45.047	46.489	46.498	47.863	48.056	50.426	50.532	53.418	52.380	45.229	51.717	7.742	7.742
	0.2	208.68	208.68	215.82	215.27	222.69	222.99	235.91	236.82	255.34	251.27	234.63	262.16	32.15	283.86

## Data Availability

Not applicable.
